# Enteric Fever Complicating Pregnancy

**Published:** 1895-08

**Authors:** G. A. Himmelsbach

**Affiliations:** Buffalo, N. Y., Clinical Instructor in medicine, University of Buffalo; Assistant Visiting Physician to the Buffalo General Hospital.


### ENTERIC FEVER COMPLICATING PREGNANCY.

By G. A. HIMMELSBACH, M. D., Buffalo, N. Y.,

Clinical Instructor in medicine, University of Buffalo; Assistant Visiting Physician to the Buffalo General Hospital.

IN THE *Medical News* of May 18, 1895, I reported a case of enteric fever with recovery, complicated by pregnancy and treated according to the Brand method. The following clinical memoranda record two more similar cases which have come under my observation since that report. The saying that rare cases come in groups was aptly illustrated in these cases and a remarkable coincidence was the fact that both patients were ill at the same time, lying side by side, coming from widely separated parts of the city, each apparently at about the same stage of the fever and both pregnant of nearly the same duration. I may also add that they were the only cases of typhoid fever in the hospital at the time.

CASE I.—J. H., aged 31 ; married ; had one child eleven years ago. She entered the employ of the hospital as a laundress and had worked but two weeks when she was taken suddenly ill with a sharp, stabbing pain in the lower right chest in the axillary line. Her temperature was 103° F. ; pulse, 120 ; respiration, 34. Physical examination showed diminished vesicular breathing, a few crackling râles and later a broncho-vesicular breathing. The percussion note was not much changed, had slight hacking cough but practically no expectoration. Dry cups were applied several times to the affected area, a wadded pneumonia jacket put on and a mild expectorant mixture given internally. Frequent observations were made, but at no time was I able to make out any definite area of consolidation. About the fifth day I noticed a few scattering petechiæ on the abdomen and chest and on more minute examination I found some meteorism, a slight tenderness in the right iliac region, constipation, heavily coated tongue, dry fissured lips, a peculiar breath, epistaxis and slight headache. Diagnosis: typhoid fever with a broncho-pneumonic complication. Dr. Hopkins saw the patient and confirmed the diagnosis. She was four months pregnant, but notwithstanding this, the treatment of cold plunge baths, when the temperature reached 102½° F., as suggested by Brand, was instituted immediately. The pyrexia responded promptly and effectually to the plunges, only seven in all were given and in a short time cold sponge baths supplanted the tub. The fever ran only a moderately severe course, ranging from 101° to 104.6° F., until the twentieth day, when a change in the temperature curve and a general improvement was appreciable. Convalescence uninterrupted. Discharged July 13th.

CASE II.—A. J., aged 20 ; married one and one-half years. She said she had felt tired and languid for some time past, but was confined to bed only seven days prior to her entering the hospital. Upon admission her temperature was 104° F. ; pulse, 144 ; respiration, 28. Urine, twenty-four hour specimen, 470 cc., turbid, acid reaction, offensive ; specific gravity, 1,027 ; urea, 12.69 grams ; no albumin, no sugar. Physical examination of the lungs and heart, negative. She complained of difficulty of deglutition, the cervical and sub-maxillary lymphatic glands were markedly swollen on both sides. The tonsils were not hypertrophied, but the posterior buccal wall was roughened and congested and gave the appearance of a post-diphtheritic throat. The abdomen was somewhat tympanitic, the percussion note over the cecum resonant, the area of splenic dulness increased. Deep, but gentle, pressure in the right iliac region was responded to by tension of the muscles and evidence of tenderness. She complained of daily headaches, particularly when the pyrexia was high, bowels constipated, tongue parched and heavily coated, teeth covered with sordes, lips dry and cracked, some twitching of the muscles and picking at the bed clothes. She had a few scattering spots on the abdomen and chest, but they were not the typical rose-colored spots of typhoid. She had not menstruated in three months, and this coupled with the increased size of the breasts, darkened areola and presence of an abdominal tumor, assured me that I had another case of typhoid complicated with pregnancy. She was put upon the Brand treatment. Her temperature ranged from 100° to 104.5° F. until the fifteenth day of her illness. After five or six days, plunging was discontinued. Convalescence uneventful. Bimanual examination now showed the presence of a gravid uterus of four months.

It will be observed from the foregoing reports that neither of these cases presented the regular stereotyped picture of enteric fever. The former was not only pregnant, but also had the pneumonic complication. The latter was pregnant between three and four months and had the cervical and sub-maxillary swelling which was probably metastatic.

*Conclusion.*—Although these three cases are by no means sufficient to base reliable conclusions upon, yet it is a curious fact that in these cases of typhoid fever complicated with pregnancy of from three to five months and treated with plunge baths, all presented at the onset a severe and grave type of the fever; all responded quickly to the baths *without in any way interfering with the pregnant state.* In all, the symptoms improved generally and the whole course of the fever was modified. The question arises, Are the plunge baths responsible for curtailing and modifying the disease, or does the pregnant state mitigate the intensity and severity of enteric fever?

137 West Tupper Street.

---

Hypodermatic Purgatives.—Ewald, of Berlin, (*Med. Chronicle*,) maintains that a mixture of equal parts of caffeine and chloral, in a one to five solution, when administered subcutaneously in doses of fifteen minims, will act as a purgative. He employs it particularly in acute rheumatism, where it also appears to act as a sedative.—*Medical Age.*

